# Supplementary material for: Overexpression of a Rosa rugosa Thunb. NUDX gene enhances biosynthesis of scent volatiles in petunia
Source: PeerJ. 2021 Apr 2;9:e11098. doi: 10.7717/peerj.11098 (PMC8020868; doi:10.7717/peerj.11098)
Supplement: Supplemental Information 1 [file peerj-09-11098-s001.pdf]

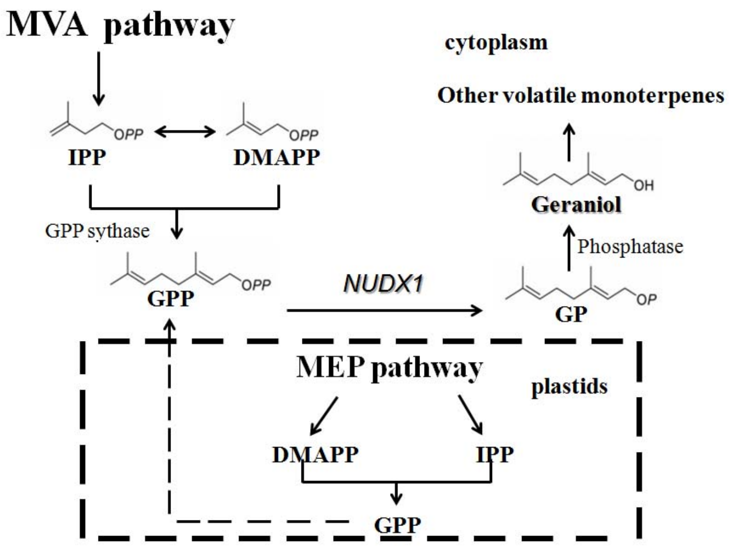

Figure S1. Hypothetical metabolic pathway of geraniol synthesis regulated by *NUDX1* gene in *Rosa×hybrida* . MVA, mevalonate; IPP, isopentenyl diphosphate; DMAPP, dimethylallyl diphosphate; GPP, geranyl diphosphate; GP, geranyl monophosphate; MEP, methylerythritol 4-phosphate (Tholl, D., and Gershenzon, J. 2015. The flowering of a new scent pathway in rose. SCIENCE 349:28-29.)

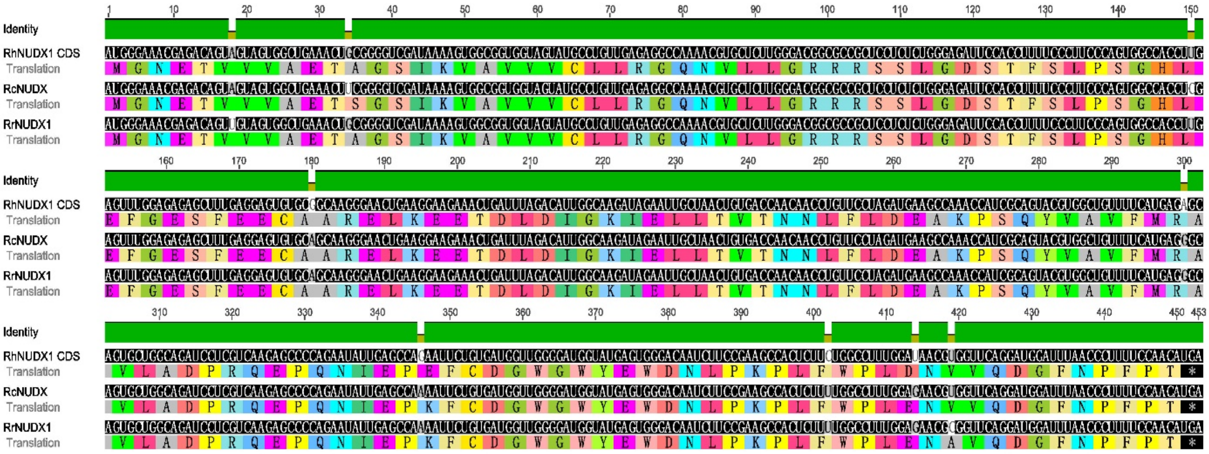

Figure S2. NUDX gene and protein sequence differences in *Rosa×hybrida* 'Papa Meilland'(Rh), *Rosa chinensis* (Rc) and *Rosa rugosa* (Rr)

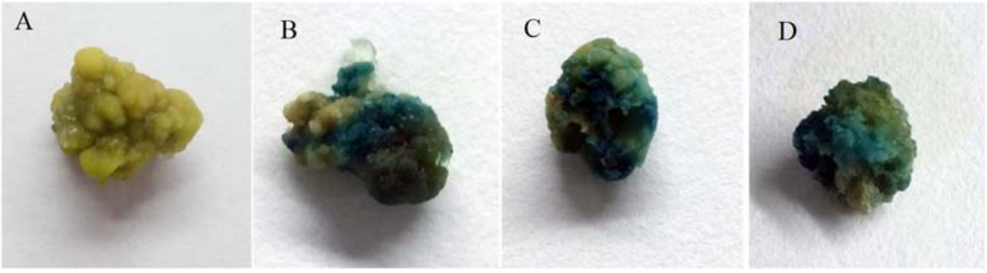

Figure S3. GUS staining results of *Petunia hybrida* callus.

A: WT; B-D: Callus with *RrNUDX1* gene transformed

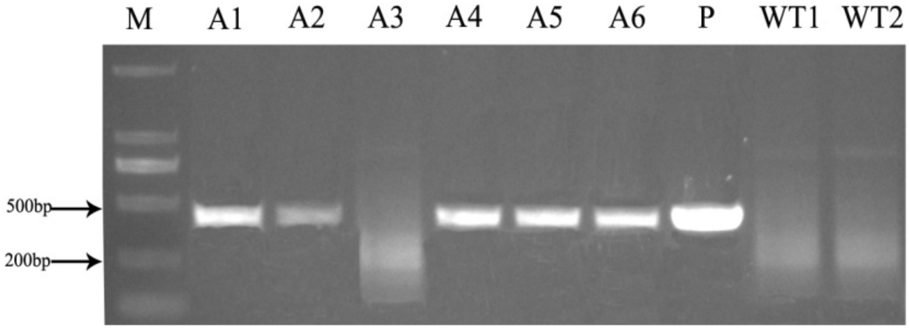

Figure S4. PCR verification of transgenic Petunia

M: Marker DL2000; A1-A6: overexpressing resistant plants; P: pCAMBIA1304-*RrNUDX1* plasmid; WT1-2: Wild type

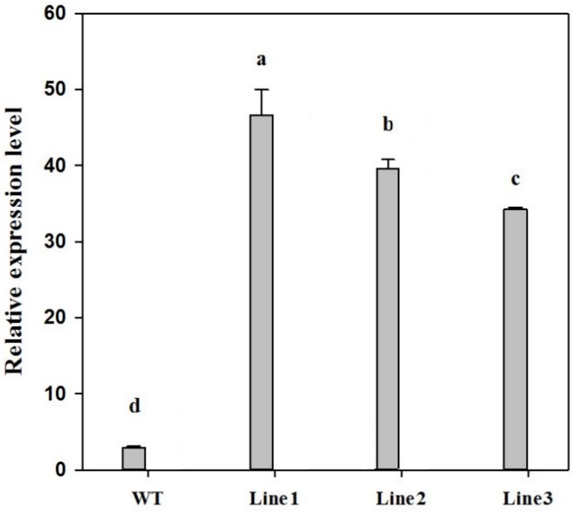

Figure S5. Expression detection of *RrNUDX1* gene in transgenic Petunia. Different letters (a, b, c) stand for significantly different (LSD test, $P < 0.05$)
